# Exploring grape marc as trove for new thermotolerant and inhibitor-tolerant *Saccharomyces cerevisiae* strains for second-generation bioethanol production

**DOI:** 10.1186/1754-6834-6-168

**Published:** 2013-11-29

**Authors:** Lorenzo Favaro, Marina Basaglia, Alberto Trento, Eugéne Van Rensburg, Maria García-Aparicio, Willem H Van Zyl, Sergio Casella

**Affiliations:** 1 Department of Agronomy Food Natural resources Animals and Environment (DAFNAE), University of Padova, Agripolis, Viale dell’Università 16, 35020 Legnaro, Italy; 2 Department of Process Engineering, University of Stellenbosch, Private Bag X1, Matieland, 7602 Stellenbosch, South Africa; 3 Department of Microbiology, Stellenbosch University, Private Bag X1, Matieland, 7602 Stellenbosch, South Africa

**Keywords:** Lignocellulosic ethanol, Grape marc, Robust yeast, Thermotolerance, Inhibitor tolerance, Sugarcane bagasse, Steam pretreatment

## Abstract

**Background:**

Robust yeasts with high inhibitor, temperature, and osmotic tolerance remain a crucial requirement for the sustainable production of lignocellulosic bioethanol. These stress factors are known to severely hinder culture growth and fermentation performance.

**Results:**

Grape marc was selected as an extreme environment to search for innately robust yeasts because of its limited nutrients, exposure to solar radiation, temperature fluctuations, weak acid and ethanol content. Forty newly isolated *Saccharomyces cerevisiae* strains gave high ethanol yields at 40°C when inoculated in minimal media at high sugar concentrations of up to 200 g/l glucose. In addition, the isolates displayed distinct inhibitor tolerance in defined broth supplemented with increasing levels of single inhibitors or with a cocktail containing several inhibitory compounds. Both the fermentation ability and inhibitor resistance of these strains were greater than those of established industrial and commercial *S. cerevisiae* yeasts used as control strains in this study. Liquor from steam-pretreated sugarcane bagasse was used as a key selective condition during the isolation of robust yeasts for industrial ethanol production, thus simulating the industrial environment. The isolate Fm17 produced the highest ethanol concentration (43.4 g/l) from the hydrolysate, despite relatively high concentrations of weak acids, furans, and phenolics. This strain also exhibited a significantly greater conversion rate of inhibitory furaldehydes compared with the reference strain *S. cerevisiae* 27P. To our knowledge, this is the first report describing a strain of *S. cerevisiae* able to produce an ethanol yield equal to 89% of theoretical maximum yield in the presence of high concentrations of inhibitors from sugarcane bagasse.

**Conclusions:**

This study showed that yeasts with high tolerance to multiple stress factors can be obtained from unconventional ecological niches. Grape marc appeared to be an unexplored and promising substrate for the isolation of *S. cerevisiae* strains showing enhanced inhibitor, temperature, and osmotic tolerance compared with established industrial strains. This integrated approach of selecting multiple resistant yeasts from a single source demonstrates the potential of obtaining yeasts that are able to withstand a number of fermentation-related stresses. The yeast strains isolated and selected in this study represent strong candidates for bioethanol production from lignocellulosic hydrolysates.

## Background

The depletion of fossil fuels together with increased environmental awareness has resulted in a strong drive towards developing eco-friendly biofuel technologies. Currently, the major alternative fuel is bioethanol, most of which is obtained from maize, wheat, and sugarcane [1-3]. However, the use of such starch-based and sugar-based materials remains controversial, because of its alternative uses for animal feed or as a staple diet of humans [1]. Ideally, the raw substrate for bioethanol production should be non-edible biomass, such as energy crops, spruce or birch, or agricultural by-products, including grain residues and sugarcane bagasse [2-5].

Lignocellulosic polysaccharides are embedded in a recalcitrant and complex matrix that requires pretreatment in order to obtain fermentable sugars. One of the most frequently used pretreatment methods is steam explosion, catalyzed by H_2_SO_4_ or SO_2_, followed by enzymatic hydrolysis to convert cellulose to glucose [3]. However, during pretreatment, the lignocellulosic material is often degraded to inhibitory compounds, such as furans, weak acids, and phenolics, which are toxic to microbial metabolism. These inhibitors have been shown to slow down or even stop the fermentation, undermining the feasibility of the process [6,7].

A variety of detoxification strategies, including alkali or sulfite treatment, evaporation, anion exchange, and laccase addition have been developed to remove these inhibitors from lignocellulosic hydrolysates or to decrease their level. However, such methods raise two key concerns regarding their technological and economic feasibility, namely the addition of costly process steps and the loss of fermentable sugars [8-10]. Therefore, several measures have been proposed as alternatives to detoxification in order to alleviate the challenges associated with inhibitors. Because the concentrations of toxic compounds and sugars in hydrolysates depend on the starting materials and on the conditions during pretreatment and hydrolysis [10,11], less recalcitrant feedstock can be selected, and mild pretreatment conditions can be applied [4,11]. Alternatively, a number of avenues to make conditions more favorable for the fermenting microorganism has been explored. The use of large inoculum has also been shown to decrease the effects of inhibition, but is considered impractical on an industrial scale [12].

In cases in which hydrolysates with high inhibitor content [13] or synthetic media supplemented with inhibitors [14] have to be used, long-term microbial adaptation to inhibitors, especially in relation to mutagenesis, represents an interesting option. The evolutionary adaptation of engineered yeasts has proven to be a powerful strategy, but often results in the loss of other desirable traits. For instance, Koppram *et al*. [9] reported that an evolutionary engineering approach enhanced the tolerance of xylose-metabolizing recombinant yeast to inhibitors derived from spruce hydrolysate, but that some of the strains lost their ability to convert xylose into ethanol.

Genetic engineering offers another means to develop highly tolerant microbes, such as in the case of *Saccharomyces cerevisiae* strains engineered to overexpress enzymes, transcription factors, and/or multidrug-resistance proteins that confer improved resistance to different inhibitors [10]. However, laboratory strains have been used for the majority of this research, and such strains may be difficult to use in industrial processes because of their generally low industrial fitness and fermenting abilities [15-17].

An alternative approach is to select for yeast strains with native resistance to inhibitors. Such a system could serve as a platform for engineering the ability of yeasts to utilize xylose or arabinose as a carbon source for ethanol production. Using naturally robust strains prevents interference with cloned genetic material, as could be the case when recombinant strains are subjected to hardening techniques.

Although many quality reports have dealt with the pretreatment of lignocellulosic materials tailored to maximize sugar release from the feedstock [2,11], very few considered yeast strains based on their innate resistance, fermentation traits, and adaptability for industrial scale [4,18]. In addition, previous screening or selection studies for tolerant *S. cerevisiae* yeasts have been targeted mainly at individual stresses, such as high temperature [19], or resistance to weak acids or furans and to phenolics [15,17], whereas finding and identifying yeasts with tolerance to multiple stresses has apparently received little attention. However, employing naturally tolerant *S. cerevisiae* would, in fact, be a more realistic approach towards developing a second-generation bioethanol industry, because it is the combined effect of the stresses that pose the greatest challenge to the success of industrial cellulosic ethanol production [20,21].

Here, we used an integrated approach with the aim of selecting new *S. cerevisiae* strains able to cope with a broad range of lignocellulose-derived fermentation inhibitors. To search for robust, thermotolerant, and strong fermenting yeasts, grape marc was assessed as this is considered an extreme environment because it has a limited availability of nutrients (such as nitrogen and carbon), it is exposed to solar radiation and to temperature fluctuations (between 20 and 45°C), has low pH, and contains ethanol and weak acids [22]. Favaro and colleagues recently described grape marc as a promising source of yeast strains with potential biotechnological applications because of their interesting extracellular enzymes [22]. However, to date, this peculiar habitat has not been considered as a possible source of novel *S. cerevisiae* yeasts with superior traits that might be exploited for second-generation bioethanol production.

Using a temperature of 40°C as a key selection criterion, a new collection of yeasts isolated from grape marc was first evaluated for their fermentation ability, as measured by their glucose consumption and ethanol production in a minimal medium supplemented with high concentrations of glucose (100 g/l) and xylose (50 g/l). Subsequently, the yeasts were screened for their inhibitor tolerance using defined broth supplemented with various concentrations of single inhibitors or cocktails of inhibitory compounds. The effect of the culture pH and sugar levels on the inhibitor tolerance of the yeasts was also addressed. Because the ultimate goal is to produce industrial yeast strains with a high fermentation capacity, hydrolysate from steam-pretreated sugarcane bagasse was used as substrate to simulate the industrial environment as closely as possible.

## Results and discussion

### Isolation and screening for efficient fermenting yeasts exhibiting thermotolerance and osmotolerance in a minimal medium

Although higher temperature fermentation is thought to be an essential phenotypic trait to maximize the efficiency of bioethanol production by yeast on a large scale, few screening surveys have thus far been conducted to search for yeasts with the ability to grow and ferment at or above 40°C [19]. With this aim in mind, we performed yeast isolations using WL (Wallerstein Laboratory) plates incubated at 38°C, 40°C, and 42°C to select for thermotolerant and robust yeasts from grape marc, which is an unexplored source of microbial biodiversity to be exploited for lignocellulosic bioethanol. As there were a large number of colonies seen at 38°C and limited growth at 42°C, colonies from plates incubated at 40°C were selected for the isolation of thermotolerant strains for further study and genotyping. All the 40 isolates were identified as *S. cerevisiae*, and were first screened for their ability to consume glucose at 40°C in must nutrient synthetic (MNS) minimal medium supplemented with either 200 g/l glucose or a combination of glucose (100 g/l) and xylose (50 g/l). These carbon sources were considered to be representative of the hexose and pentose content in most lignocellulosic hydrolysates [3,23]. In this work, the ability of the yeasts to consume glucose was defined as the fermenting vigor and expressed in terms of grams of glucose consumed per liter of MNS broth, as described in ‘Methods’.

Owing to their relatively diverse phenotypic backgrounds, five control strains of *S. cerevisiae* were included in this study as benchmarks. Three of these *S. cerevisiae* benchmark strains (MH1000, DSM70449 and 27P) have been used previously for ethanol production from different lignocellulosic substrates [24-28], and the oenologically relevant *S. cerevisiae* EC1118 and the laboratory strain Y294 were included as additional benchmarks.

**Figure 1:**
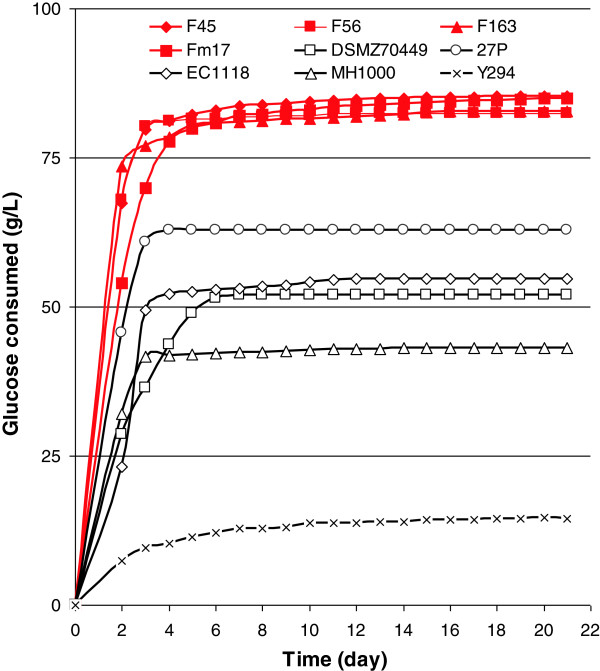
**Cumulative sugar utilization (grams of glucose consumed per liter of MNS) of selected *Saccharomyces cerevisiae *isolates and reference yeast strains.** Strains were incubated at 40°C in MNS medium with 100 g/l glucose and 50 g/l xylose. All experiments were conducted in triplicate, with the relative standard error always being less than 5% (not reported).

To assess the fermenting vigor of the 40 isolates, cultures were incubated at 40°C and 25°C, with the latter serving as the temperature control. Generally, the isolates exhibited a high and comparable level of fermenting vigor in relation to the results achieved by the control strains, (Figure 1). When yeasts were incubated at 40°C in MNS with 100 g/l glucose and 50 g/l xylose (Figure 1), the *S. cerevisiae* isolates F45, F56, F163, and Fm17 displayed the greatest degree of glucose consumption, much higher than that achieved by the reference yeasts. For example, the degree of glucose consumed by isolate Fm17 was more than five-fold greater than that of the weakest control strain, Y294, and 1.3-fold higher than the best control strain, 27P.

The performance of the remaining yeast isolates was better than that of the benchmark strain, 27P (data not shown). By contrast, the other benchmark yeasts generally exhibited a low capability to withstand higher temperatures and osmotic stress, as they consumed only up to 50 g/l glucose, with the laboratory strain, Y294, showing the poorest fermenting vigor.

### In-depth assessment of four selected isolates in a minimal medium

Owing to the large volume of data generated from the high-performance liquid chromatography (HPLC) assessment of the 40 isolates, the four best fermenting isolates of *S. cerevisiae* were selected for further investigation in terms of their consumption of the carbon source and production of ethanol and by-products (Table 1). The performance of the four isolates, designated as strains F45, F56, F163, and Fm17 were compared with the four benchmark strains 27P, MH1000, EC1118, and DSM70449. The laboratory strain, Y294, which exhibited poor fermentation vigor, was not included in this assessment.

**Table 1:** **Sugar consumption and product formation by the best fermenting *S. cerevisiae *** **isolates and benchmark strains**^**a**^

	***S. cerevisiae *** **strains**							
	**27P**^**b**^	**EC1118**^**b**^	**MH1000**^**b**^	**DSM70449**^**b**^	**F45**^**c**^	**F56**^**c**^	**F163**^**c**^	**Fm17**^**c**^
25°C								
Glucose, g/l	5.1	7.0	8.1	14.8	3.7	1.0	5.2	4.1
Xylose, g/l	48.2	45.0	46.3	46.7	44.0	46.5	47.9	46.5
Xylitol, g/l	1.7	4.8	3.5	2.9	5.8	3.4	2.0	3.3
Glycerol, g/l	3.6	3.9	3.7	3.6	3.2	3.3	3.1	3.0
Ethanol, g/l	43.8	43.4	40.6	34.9	44.0	46.5	44.9	46.1
Ethanol yield^d^	0.46	0.47	0.44	0.41	0.46	0.47	0.47	0.48
Ethanol yield, %^e^	90	92	87	80	90	92	93	94
Glycerol yield^f^	0.038	0.042	0.040	0.042	0.033	0.033	0.033	0.031
40°C								
Glucose, g/l	38.1	43.4	49.2	47.8	16.0	18.5	16.6	15.0
Xylose, g/l	47.4	46.7	48.3	46.3	48.7	48.3	49.4	48.3
Xylitol, g/l	2.2	2.1	2.8	2.8	4.0	3.8	3.8	2.6
Glycerol, g/l	3.0	2.5	2.7	2.8	2.6	2.7	2.8	2.7
Ethanol, g/l	25.5	22.7	18.5	19.5	37.8	38.0	38.1	39.2
Ethanol yield^d^	0.41	0.40	0.36	0.37	0.45	0.47	0.46	0.46
Ethanol yield, %^e^	81	79	71	73	88	91	90	90
Glycerol yield^f^	0.048	0.044	0.053	0.054	0.032	0.033	0.034	0.032

^a^Measured after 21 days fermentation at 25 and 40°C in MNS broth supplemented with glucose (100 g/l) and xylose (50 g/l). All experiments were conducted in triplicate, and relative standard error was always less than 5% (not reported).

^b^Control strains.

^c^*S. cerevisiae *isolated from grape marc.

^d,e^Ethanol yield as ^d^grams of ethanol per gram of consumed glucose and ^e^percentage of theoretical maximum (0.51 g/g from glucose).

^f^Glycerol yield as grams of glycerol per gram of consumed glucose,

At 25°C, the selected strains produced ethanol levels comparable with those of the benchmark yeasts, with strains F163 and Fm17 exhibiting the highest ethanol yields (Table 1). At 40°C, the selected strains produced ethanol concentrations ranging between 37.8 and 39.2 g/l, where the latter corresponded to an ethanol yield equal to 91% of the theoretical maximum (defined as fermenting efficiency), whereas the reference strains had significantly lower fermenting efficiencies, with *S. cerevisiae* 27P being the most efficient strain, having an ethanol yield of 81% of the theoretical maximum. Ethanol yields of the selected strains at 40°C were comparable with those achieved in MNS supplemented with 200 g/l glucose and no xylose (92%, 94%, 92%, and 91% of the theoretical yield for strains F45, F56, F163, and Fm17, respectively).

In general, half of the supplied glucose remained in the broth at the end of the fermentation by the benchmark yeasts, indicating ethanol inhibition, which is known to increase with temperature [19].

Generally, no xylose consumption was detected, and only small amounts of xylose were reduced to xylitol (Table 1). The low level of xylose reduction suggested that the isolated yeasts might have limited xylose reductase capabilities, although non-specific aldose reductase activity might also have contributed to the low levels of the detected xylitol, which could not be oxidized to xylulose, possibly because of co-factor imbalances. This hypothesis is consistent with previous work describing xylose reduction in wild type *S. cerevisiae* strains [29,30].

Compared with the control strains, the selected yeasts exhibited interesting behavior in terms of glycerol production in response to the harsh culture conditions (Table 1). At 25°C*,* this metabolic by-product was produced at levels in the range of 6 to 10% of the ethanol concentration, suggesting that all strains had an efficient glucose to ethanol conversion pathway [31]. However, an increase in the temperature to 40°C resulted in a marked increase in glycerol concentration relative to ethanol concentration for the reference strains, whereas this ratio remained unchanged for the selected strains. This lack of a glycerol response in the selected strains was also evident in the yield of glycerol from the consumed glucose, which remained comparable at both incubation temperatures, but was markedly lower than that of the reference strains at 40°C (Table 1). Similarly, with the reference strains, an increase in temperature resulted in a decrease of up to 18% in the ethanol yield from the consumed glucose, whereas this decrease was no more than 4% in the case of the selected strains. These data clearly indicate a higher degree of tolerance to heat stress in the selected strains, as evident from their lower glycerol yield and improved ethanol yield relative to the control strains, under harsh conditions. The two most important functions of glycerol synthesis in yeast have been previously related to redox balancing and the hyperosmotic stress response [31]. Our findings suggest that glycerol may have several additional roles in the complexity of the microbial metabolism related to multiple environmental stress tolerance, suggesting that glycerol production is a strain-specific trait. A similar hypothesis was previously proposed by Ribereau-Gayon *et al*. [32], who suggested that glycerol production in *S. cerevisiae* might be a strain-related strategy to withstand high temperature.

The high glucose consumption and ethanol yield achieved by the selected yeasts at 25°C and 40°C might also be ascribed to their greater degree of osmotolerance as compared to the reference yeasts (Table 1). This hypothesis is consistent with previous researches on osmotolerant *S. cerevisiae* strains exhibiting high glucose consumption rates and ethanol yields in the presence of higher sugar concentrations [33-35]. The extent of osmotic tolerance in the newly isolated yeasts will need to be quantified in future studies.

Overall, the fermentation parameters exhibited at 40°C by this new collection of yeasts isolated from grape marc were markedly better than those reported in previous studies [19]. Hacking *et al*. [36] screened a total of 55 yeast strains for glucose fermentation at higher temperatures, and achieved yields of 50% of the theoretical maximum with 12 strains cultured at 40°C. Thermotolerant yeast strains have additionally been isolated from hot climates or regions. A noteworthy screening was performed by Pellegrini and colleagues, who reported that, out of 457 *S. cerevisiae* cultures, DBVPG 1849, isolated from Ethiopian wine, was the most efficient fermenting strain at 40°C, with an ethanol yield of nearly 85% of theoretical maximum [37]. Given that, at 40°C, DBVPG 1849 has the highest glucose to ethanol conversion yield of any strain described to date, our collection of strains, with ethanol yields of up to 94% of theoretical maximum, exhibit outstanding ethanol conversion performance at the same high temperature. Therefore, to our knowledge, this is the first account describing *S. cerevisiae* strains capable of fermenting glucose at 40°C with ethanol yields close to 94% and 91% of theoretical maximum in the presence of either 200 g/l glucose, or 100 g/l glucose plus 50 g/l xylose, respectively. In addition, because thermotolerance in *S. cerevisiae* strains has thus far been screened by incubating the strains in complex media, such as YPD (yeast peptone dextrose) or similarly formulated broths [19,36,37], the fermenting abilities of the strains selected in this study are even more significant, given that they were achieved in MNS minimal broth and that the fermentations were based on a low initial inoculum size (about 10^5^ cells per ml).

### Inhibitor tolerance in defined medium

We also compared the growth data of the *S. cerevisiae* isolates, together with the five benchmark strains, in YNB (yeast nitrogen base) medium, in the presence of increasing concentrations of inhibitory compounds (weak acids and furans), formulated as single toxic components or combined in inhibitor cocktails. For each strain, the tolerance was evaluated as relative growth (optical density (OD) value, %) by comparing the yeast growth in the medium containing inhibitory compound(s) with that in medium lacking these compound(s).

In addition to the combination of glucose (100 g/l) and xylose (50 g/l) used in the initial screen, culture growth was also assessed at a glucose concentration of 20 g/l to screen for yeasts capable of withstanding inhibitors at sugar levels similar to those in most lignocellulosic fermentations [2,3]. In all experiments, performed using YNB supplemented with 20 g/l glucose, the acidity of the medium was adjusted to pH 4.5, and the performances of the six most promising isolates, selected on the basis of their tolerance to each toxic compound, and of *S. cerevisiae* 27P (the most tolerant benchmark yeast) were assessed (Table 2).

**Table 2:** **Influence of weak acids (acetic and formic acid) and furans (furfural and HMF) on growth in YNB medium (supplemented with glucose 20 g/l) pH 4.5, of the most inhibitor-tolerant newly isolated *S. cerevisiae *** **strains and the most resistant benchmark yeast 27P**^**a**^

**Inhibitor**	**Concentrations**		***S. cerevisiae *** **strains**						
	**mmol/l**	**g/l**	**27P**^**ab**^	**Fm12**	**Fm17**	**Fm38**	**Fm64**	**Fm89**	**Fm90**
Lactic acid	19	1.72	**100**	**100**	**100**	**100**	**100**	**100**	**100**
	38	3.45	**100**	**100**	**100**	**100**	**100**	**100**	**100**
	57	5.17	**100**	**100**	**100**	**99**	**100**	**100**	**100**
	76	6.89	**99**	**100**	**100**	**96**	**100**	**100**	**100**
Formic acid	13	0.61	**99**	**91**	**94**	**90**	**92**	**94**	**93**
	27	1.22	**93**	**91**	**94**	**90**	**92**	**91**	**91**
	40	1.83	89	89	**91**	88	**90**	89	89
	53	2.44	85	86	**90**	83	87	87	86
Acetic acid	30	1.80	**99**	**96**	**99**	**95**	**98**	**96**	89
	60	3.60	89	**90**	**96**	87	**96**	**92**	88
	90	5.40	86	88	**92**	84	89	**90**	83
	120	7.20	82	87	**91**	78	88	85	80
Furfural	7	0.69	**92**	**90**	**93**	**94**	**95**	**90**	**90**
	14	1.38	88	84	89	**90**	**91**	85	74
	22	2.08	67	77	86	61	87	58	52
	29	2.77	*12*	*0*	60	*28*	51	*39*	*29*
HMF	7	0.94	87	**92**	**91**	**90**	82	87	**91**
	15	1.86	84	90	81	80	77	70	87
	22	2.81	73	84	78	75	69	59	79
	30	3.75	*48*	74	73	64	*35*	*48*	70
Cocktail^c^									
A	–	–	83	80	**91**	**90**	87	88	82
B	–	–	65	70	80	70	70	72	63
C	–	–	*35*	51	71	55	63	60	52
D	–	–	*0*	*0*	*0*	*0*	*0*	*0*	*0*

*Abbreviations*: *HMF *5-hydroxymethylfurfural, *YNB *yeast nitrogen base.

^a^Values are reported as relative growth (%) of the optical density measured for each strain after 40 hours of growth in YNB without inhibitor, and are the means of three replicates. Standard error was always less than 4% (not shown). Bold and italic fonts are used for values equal to or greater than 90 and equal to or less than 50, respectively.

^b^Control strain.

^c^For compostion of inhibitor cocktails, please see Table 3.

On a molar basis, formic acid was more toxic than acetic acid, as the highest concentration of formic acid (53 mmol/l) produced inhibitory effects similar to those seen with 120 mmol/l acetic acid. Accordingly, when exposed to the highest dose of both acids, the yeasts showed relative growth values ranging from 80% to 91% of the culture growth achieved in medium without acids, with strains Fm12, Fm17, Fm64, and Fm89 showing the most promising results. Conversely, increases in lactic acid had little apparent effect on culture growth, which is consistent with the literature [16]. Furthermore, the performance of the control *S. cerevisiae* 27P exhibited a similar trend, although the values for this strain in the presence of individual weak acids were at the bottom end of the range of values recorded for the other cultures.

Of the furans, furfural was the most toxic, as evident from the 30% decrease in relative growth on average, observed with 2.08 g/l furfural for the selected yeasts, although strains Fm17 and Fm64 exhibited the greatest degree of tolerance at 2.77 g/l furfural. Similarly, supplementation with 5-hydroxymethylfurfural (HMF) also resulted in severe decreases in growth, although these responses were not as dramatic as for furfural. In the presence of 2.81 g/l HMF, the yeasts showed relative growth values ranging from 59% to 84% of the culture growth achieved in the medium without this inhibitor, with strains Fm12, Fm17, and Fm90 showing the highest level of tolerance also at 3.75 g/l HMF.

Inhibitor cocktails, formulated as described in Table 3, severely hindered cell growth (Table 2), with the benchmark yeast being the most sensitive strain. Although cocktails A and B generally resulted in strong growth inhibition, cocktails C and D had the highest negative effects on yeast growth. Nevertheless, strain Fm17 exhibited the highest degree of tolerance, with a relative growth value of 71%. By contrast, cocktail D (formulated with acetic acid 7.20 g/l, formic acid 2.44 g/l, lactic acid 6.89 g/l, furfural 2.77 g/l and HMF 3.75 g/l), did not support any growth of any of the strains tested, suggesting that each of the inhibitory compounds within the cocktail may have synergistically challenged the yeasts to grow under these multiple environmental stresses.

**Table 3:** Composition of synthetic inhibitor cocktails added to supplemented YNB broth

**Inhibitor**	**Cocktail**			
	**A**	**B**	**C**	**D**
Acetic acid, g/l	1.80	3.60	5.40	7.20
Formic acid, g/l	0.61	1.22	1.83	2.44
Lactic acid, g/l	1.72	3.45	5.17	6.89
Furfural, g/l	0.69	1.38	2.08	2.77
HMF, g/l	0.94	1.86	2.81	3.75

*Abbreviations*: *HMF *5-hydroxymethylfurfural.

Overall, the promising inhibitor-tolerant phenotypes detected in YNB at pH 4.5 seem to be notable compared with the relevant literature. Many previous reports on *S. cerevisiae* inhibitor endurance have mainly used complex YPD-based broths or defined media similar to YNB, adjusting the pH at higher values (up to 6.5) [9,16]. As a result, the higher pH values may have *de facto* decreased the strong inhibiting power of the aliphatic acids to which the cultures were exposed. In addition, in order to identify robust yeasts, the current study was specifically designed to screen for the inhibitor tolerance of yeasts with a starting inoculum size (about 10^6^ cells per ml) of about 10 times lower than those normally used for similar experimental activities [17,38].

In order to enhance the environmental stresses that the yeasts had to be able to withstand, YNB was supplemented with inhibitor cocktails together with high concentrations of sugars (100 g/l glucose and 50 g/l xylose). Of the selected strains, *S. cerevisiae* Fm17 proved to be the most robust under these conditions, with a relative growth value of nearly 85% in cocktail C. Consequently, this strain was chosen as the most tolerant newly isolated yeast for further fermentation trials using synthetic cocktails and lignocellulosic hydrolysate.

### Fermentation performance of *S. cerevisiae* strains Fm17 and 27P (benchmark) in YNB supplemented with inhibitor cocktails

The ethanol production of *S. cerevisiae* Fm17 and the benchmark yeast 27P was compared in YNB supplemented with inhibitor cocktails (Table 4), and the combination of glucose (100 g/l) and xylose (50 g/l). Strains Fm17 and 27P, which exhibited high ethanol yields at 25°C and 40°C (Table 1), were selected as the most inhibitor-tolerant isolated and benchmark strains (Table 2). Because we had found Fm17 to be one of the most thermotolerant of the initial 40 yeast isolates (Figure 1, Table 1), these experiments were conducted at 30°C, which was closer to the optimum temperature of the reference yeast, to better illustrate differences in performance by these two strains. Both yeasts were first evaluated for their ability to ferment in the presence of the cocktails A, B, C, and D, formulated by adding increasing concentrations of each inhibitory compound as described earlier (Table 3). In the presence of cocktails A and B, the fermentation performance of the yeast strains was similar, with their volumetric productivities and glucose consumption rates being generally greater than those recorded in the reference medium (without inhibitor supplementation) (Table 4). This is probably attributable to the presence of weak acids, which are known to enhance fermentation rate at low concentrations (below 100 mmol/l) [10]. By contrast, in cocktail C, which had a total weak acids content close to 187 mmol/l, Fm17 achieved a volumetric productivity comparable with that of the control supplemented YNB broth, whereas the productivity of the reference strain, 27P, was two-fold lower than in the broth without inhibitors and was also two-fold lower than that of strain Fm17. The tolerance of Fm17 was even more pronounced in cocktail D, formulated with the highest inhibitor levels (Table 4). The ethanol levels reached 19 g/l although the specific productivity of 0.11 g/g/h was three-fold lower than that detected in the supplemented YNB broth without inhibitors.

**Table 4:** **Effects of synthetic inhibitor cocktails and sugarcane hydrolysate formulations supplied at different concentrations on the fermentation performance at 30°C of the newly isolated *S. cerevisiae *** **strain, Fm17, and the benchmark *S. cerevisiae *** **strain, 27P, when incubated in the presence of 100 g/l glucose and 50 g/l xylose**^**a**^

**Strain**	**Inhibitor cocktail**^**b**^	**Highest ethanol concentration, g/l**	***Y***_**E/G**_, **g/g**	***Q***_**48h**_, **g/l/h**	***q***_**48h**_, **g/g/h**	**Y**_**X/G**_, **g/g**	**Glucose consumption rate at 48 h, g/l/h**
Fm17	None	49.4	0.49 (97%)	0.88	0.34	0.027	1.73
	A	48.6	0.49 (95%)	1.01	0.31	0.030	2.07
	B	47.9	0.48 (94%)	1.00	0.30	0.031	2.06
	C	47.1	0.47 (92%)	0.87	0.31	0.030	1.89
	D	19.0	0.46 (90%)	0.07	0.11	0.009	0.24
27P	None	48.9	0.49 (95%)	0.88	0.29	0.026	1.68
	A	48.3	0.48 (95%)	1.02	0.30	0.029	2.07
	B	46.7	0.47 (92%)	0.97	0.31	0.029	2.04
	C	45.0	0.45 (88%)	0.43	0.22	0.027	0.92
	D	0.3	0.29 (58%)	0.01	0.02	0.003	0.02
Fm17	0% SH	48.8	0.49 (96%)	0.93^c^	ND	ND	2.24^c^
	25% SH	47.6	0.47 (92%)	1.02^c^	ND	ND	2.38^c^
	50% SH	43.4	0.45 (89%)	0.70^c^	ND	ND	1.76^c^
	75% SH	18.6	0.42 (82%)	0.22^c^	ND	ND	0.53^c^
	100% SH	–	–	–	–	–	–
27P	0% SH	47.7	0.48 (94%)	0.95^c^	ND	ND	2.28^c^
	25% SH	44.0	0.44 (86%)	1.04^c^	ND	ND	2.38^c^
	50% SH	40.6	0.42 (83%)	0.46^c^	ND	ND	1.12^c^
	75% SH	2.4	0.24 (46%)	0.03^c^	ND	ND	0.19^c^
	100% SH	–	–	–	–	–	–

ND, not determined; *Q*_48h_, volumetric productivity after 48 h; *q*_48h_, specific productivity after 48 h; SH, sugarcane hydrolysate; *Y*_E/G_, ethanol yield per gram of consumed glucose calculated on the basis of the highest ethanol production (the percentage of theoretical maximum is indicated in brackets); Y_X/G_, biomass yield after 72 hours on initial glucose.

^a^All experiments were conducted in triplicate. Any standard deviations that were always less than 5% are not reported.

^b^The combination of 100 g/l glucose and 50 g/l xylose was used to supplement YNB broth without inhibitors (reported in the table as ‘none’ or ‘0% SH’).

^c^Parameter determined after 42 hours.

**Figure 2:**
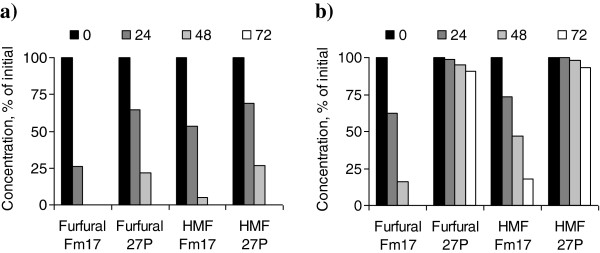
**Conversion of furfural and 5-hydroxymethylfurfural (HMF) after 0, 24, 48, and 72 hours of fermentation with *Saccharomyces cerevisiae *strains Fm17 and 27P in the presence of inhibitor cocktails. (a)** Cocktail C and **(b)** cocktail D. Experiments were conducted in triplicate. Relative standard error was always less than 4% (not reported).

The higher fermenting abilities of the selected yeast, Fm17, in cocktail C could be attributable to a more pronounced ability to convert furfural and HMF compared with the reference strain, 27P (Figure 2). The yeast strains decreased the levels of furfural before they decreased the levels of HMF, which is in line with previous work [15]. More importantly, the stronger furan tolerance phenotype of Fm17 was confirmed, as evident from the decrease of these compounds in the more toxic cocktail D (Figure 2b). After 72 hours of incubation, Fm17 reduced the furfural and HMF concentrations in the cocktail D to 9% of the initial concentrations, whereas 27p produced no significant conversion of both furans (Figure 2b). On the one hand, the fact that Fm17 converted the inhibitors more rapidly compared with 27P may indicate an enhanced ability of Fm17 to metabolize furan components. As an alternative, this more rapid conversion could be a result of a higher metabolic rate in Fm17, as suggested by the significantly higher biomass yield after 72 hours and significantly higher volumetric glucose consumption rate after 48 hours (Table 4).

Overall, Fm17 exhibited the most promising ethanol yield in all the tested cocktails, producing nearly 0.46 g ethanol per gram of glucose (90% of theoretical yield) in cocktail D, which represented the harshest conditions (Table 4). This superior performance was clearly evident compared with the control strain, 27P. Excepting in cocktail D, the biomass yields of both yeasts were greater at the end of fermentation in YNB broth containing inhibitors compared with YNB broth without inhibitor supplementation, suggesting that the furans and weak acids may have exerted a beneficial effect on biomass production.

It is noteworthy that lower amounts of glycerol and xylitol were detected for both yeasts in the presence of each inhibitor cocktail, compared with the levels seen in the control supplemented YNB without inhibitors (data not shown). Because both furfural and HMF were metabolized by both yeasts (Figure 2), whereas no difference was seen in the concentration of aliphatic acids (data not shown), it is possible that the furans might have acted as external electron acceptors during the fermentation, resulting in diminished xylitol formation. The lower glycerol production may be explained by the fact that, for yeast metabolism, reduction of furfural to furfuryl alcohol is preferred to glycerol as a redox sink [15,39].

### Fermentation performance of *S. cerevisiae* strains Fm17 and 27P (benchmark) in YNB supplemented with sugarcane bagasse hydrolysate

It is possible that the fermentation performance of yeasts is different in lignocellulosic hydrolysates and synthetic cocktails because of the hampering action of other toxic compounds that cannot be identified or quantified [2], in spite of the cocktails having the same composition in terms of the major hydrolysate inhibitors. The main goal of this work was to isolate, screen, and characterize new *S. cerevisiae* strains for second-generation industrial bioethanol production, based on their robustness and strong fermentation performance. Therefore, we used hemicellulose hydrolysate from steam-pretreated sugarcane bagasse as our source of inhibitors. This feedstock is one of the most abundant sources of lignocellulose in the world that together with steam pretreatment, which is one of the most frequently used pretreatment methods [3], would result in conditions that are representative of bioethanol production worldwide. The hemicellulose hydrolysate (hereafter referred to as ‘hydrolysate’), produced after steam pretreatment at 200°C for 10 minutes, contained low levels of sugars (mainly xylose) and relatively high concentrations of inhibitors, including 2.0 g/l furaldehydes, more than 14 g/l aliphatic acids, and considerable amounts of phenolic acids and aldehydes (see Additional file 1: Table S1). By comparison, levels of 1.5 to 1.6 g/l for furaldehydes and 5.2 to 5.5 g/l for aliphatic acids were previously detected by Martín *et al*. in two enzymatic hydrolysates of sugarcane bagasse [40]. These authors described the inability of their yeast strain to ferment a third hydrolysate containing 4.5 g/l furaldehydes and 7.4 g/l aliphatic acids.

**Figure 3:**
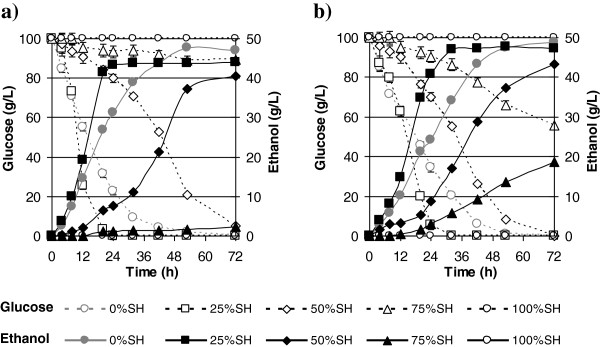
**Ethanol production and glucose consumption by yeast strains in different sugarcane hydrolysate (SH) formulations. (a)** The isolated *Saccharomyces cerevisiae* strain Fm17 and **(b)** the benchmark industrial *S. cerevisiae* 27P. The SH formulations (volume basis) were 25%, 50%, 75%, and 100% SH, and the broth was supplemented with 100 g/l glucose and 50 g/l xylose. All experiments were conducted in triplicate, and are reported as mean ± standard deviations.

To evaluate the ability of our selected yeast to ferment carbon in the presence of sugarcane bagasse hydrolysate, YNB broth was supplemented with four different concentrations of sugarcane hydrolysate (SH) to final concentrations (all on a volume basis) of 25%, 50%, 75%, and 100% SH. YNB without hydrolysate served as the control, and was designated as 0% SH. All media were supplemented with 100 g/l glucose and 50 g/l xylose as carbon sources (Figure 3).

In the presence of 25% SH, the yeasts produced comparable ethanol titers (47.6 and 44.0 g/l for Fm17 and 27P, respectively) and displayed volumetric productivities up to 1.1-fold greater than that recorded in 0% SH (Table 4). The fact that similar behavior was observed in the synthetic cocktails (Table 4) suggests that the weak acids in the hydrolysate again exerted a beneficial effect on ethanol production by both strains. The rate of glucose consumption in 25% SH was clearly greater than in the absence of SH (0% SH) (Figure 3). In 50% SH, the highest ethanol concentrations were comparable for both strains (Table 4), although Fm17 achieved a volumetric productivity that was 1.5-fold greater than that of 27P. Most importantly, the dramatic improvement in tolerance of Fm17 was clearly exemplified by its fermentation of 75% SH, producing up to 18.6 g/l ethanol, whereas the ethanol level achieved by 27P was 7.7-fold lower (Figure 3). However, no growth or ethanol production was detected in the 100% SH solution for either of the strains (Figure 3). Similar results were obtained by Martin *et al*. [40], who observed no ethanol production in the presence of undiluted H_2_SO_4_-impregnated sugarcane bagasse, in which the content of furans was two-fold greater than that of the pretreated bagasse we used (see Additional file 1: Table S1) and the concentration of weak acids was two-fold lower (7.4 instead of 14.2 g/l).

The greater degree of robustness of strain Fm17 is also evident from the data presented in Table 4. Fm17 resulted in ethanol yields of almost 0.45 and 0.42 g ethanol per gram of glucose in 50% SH and 75% SH, respectively, and these levels were significantly higher than those determined for strain 27P. The greater robustness of strain Fm17 is further exemplified when compared with the data published by Martin *et al*. using *S. cerevisiae* ATCC 96581 [41]. ATCC 96581, which was isolated from a spent sulfite liquor (SSL) fermentation plant, was grown in a medium containing a composition of weak acids, furans, and aldehydes comparable with that contained in sugarcane hydrolysate used in our work and described in Methods [41]. Although this strain also exhibited high levels of tolerance [18], the ethanol yield was only 0.28 g ethanol per gram of glucose, which was at least 1.4-fold lower than that determined for strain Fm17 in the present study. Isolation of yeasts from grape marc thus proved to be a highly efficient strategy for obtaining tolerant yeast, given the hostile environment presented by SSL.

## Conclusions

The integrated approach followed in this study, with a variety of different selective pressures imposed on *S. cerevisiae* strains and the strategic choice of grape marc as the source of tolerant yeasts, was effective in isolating new strains capable of coping with the most significant stresses prevalent in large-scale bioethanol production. Our results show that grape marc is a promising environment for the isolation of yeasts showing inhibitor, temperature, and osmotic tolerance, and these proved to be substantially more robust than the established industrial strains. The study results suggest that further unconventional ecological niches should be explored to select yeasts suitable for second-generation bioethanol production. In addition, the phenotypic differences in inhibitor tolerance between the screened yeast isolates shows that the strain selection is crucial in the design of a process involving fermentation in the presence of lignocellulosic hydrolysates. Given the strong performance of *S. cerevisiae* Fm17 described in this study, this strain should serve as an excellent platform for further genetic engineering to enhance ethanol production in terms of rate and yield through co-fermentation of all available carbon.

## Methods

### Yeast isolation, identification, and strains

The following five benchmark *S. cerevisiae* yeasts were used: *S. cerevisiae* Y294 *laboratory strain ATCC201160; ATCC, Manassas,Virginia, USA); *S. cerevisiae* DSM70449/(top fermenting beer strain; Leibniz-Institut DSMZ, Braunschweig, Germany); *S. cerevisiae* EC1118 (commercial wine yeast strain obtained from Lallemand Fermented Beverages, Castel D'Azzano Verona, Italy); *S. cerevisiae* MH1000 (industrial distillery yeast; Stellenbosch University, South Africa) and the *S. cerevisiae* 27P (industrial yeast) [26].

New yeast strains were isolated from grape marc collected during the vintage 2010, immediately after crushing, from a winery located in Melara, (Rovigo) Italy (45°4′0′N, 11°12′0″E). The grape marc contained a mixture of four different varieties, namely Prosecco (*Vitis vinifera* cv. Prosecco), Moscato (*Vitis vinifera* cv. Moscato), Raboso (*Vitis vinifera* cv. Prosecco) and Nebbiolo (*Vitis vinifera* cv. Nebbiolo).

In the laboratory, 20 g of grape marc were dispersed in 200 ml of sterile physiological saline (0.85% NaCl). After appropriate decimal dilutions, this was plated onto WL (Wallerstein Laboratory, Oxoid Limited, Basingstoke,United Kingdom) solid medium containing 100 μg/ml chloramphenicol (Sigma-Aldrich, St. Louis, USA) to prevent bacterial growth, and incubated at different temperatures (38°C, 40°C and 42°C) for 72 hours. After isolation, yeast colonies were purified by growing on yeast and mould agar medium (YM; Oxoid Limited, Basingstoke, United Kingdom) at 40°C for 48 hours. Isolates were maintained at -80°C in YM broth containing 20% (v/v) glycerol.

Genetic identification of the strains was achieved by sequence analysis of the D1/D2 region. Amplification of the D1/D2 domain was performed by PCR using primers NL1 (5′-GCATATCAATAAGCGGAGGAAAAG - 3′) and NL4 (5′-GGTCCGTGTTTCAAGACGG-3′), following the protocol described by Kurtzman and Robnett [42]. Amplification products were checked for purity by agarose gel electrophoresis and then sequenced using an ABI protocol for Taq-Dye Terminator Sequencing (Applied Biosystems, Life Technologies Corporation, Carlsbad, Ca, USA) on an automated sequencer (ABI377; Applied Biosystems, Life Technologies Corporation, Carlsbad, Ca, USA). The obtained sequences were edited with Chromas Lite (version 2.1.1; Technelysium Pty Ltd, South Brisbane, Australia), and species identification was performed by BLASTn alignment (http://blast.ncbi.nlm.nih.gov/Blast.cgi?PROGRAM=blastn&PAGE_TYPE=BlastSearch&LINK_LOC= blasthome) with sequences present in the GenBank public database. A sequence similarity level of 100% was considered to be positive species identification.

### Fermentation abilities of *Saccharomyces cerevisiae* strains in minimal broth supplemented with high sugar concentrations

In total 40 *S. cerevisiae* strains were evaluated for their fermentation ability in must nutrient synthetic (MNS) medium [43] supplemented with either 200 g/l glucose or with 100 g/l glucose and 50 g/l xylose. The latter combination was used because these are the highest reported levels of these two sugars in steam-pretreated lignocellulosic materials [3,11].

The fermentations were performed under oxygen-limited conditions in 110 ml glass bottles (working volume of 100 ml), sealed with rubber stoppers and equipped with needles for carbon dioxide removal and sampling. Pre-cultures of *S. cerevisiae* strains that had been grown to stationary phase in YPD broth were inoculated with an average concentration of 7.5 × 10^4^ cells per ml and incubated in static conditions at 25°C and 40°C. Fermentation vigor was monitored daily by measuring bottle weight loss in relation to CO_2_ production, and reported, using a conversion factor of 2.118 [43], as grams of glucose utilized per liter of MNS. The experiments were carried out in triplicate. Samples were withdrawn daily, filtered through 0.22 μm membrane filters, and analyzed for their content of glucose, xylose, xylitol, glycerol and ethanol by HPLC as described by Favaro *et al*. [5].

### Screening for inhibitor tolerance

The newly isolated yeasts and the reference strains were evaluated for their inhibitor tolerance in defined YNB broth without amino acids (Difco, Italy) supplemented either with glucose 20 g/l or with glucose 100 g/l and xylose 50 g/l and containing increasing concentrations of weak acids (acetic, formic, and lactic acids) and furans (furfural and HMF), either as single compounds or as inhibitor cocktails. The effects of pH on the inhibitor tolerance of yeast were also assessed. The pH in both media either was left unchanged or was adjusted to 4.5 after inhibitor addition, using 5 mol/l NaOH or HCl. This pH value was chosen because it is widely used in many bioethanol production processes [44,45].

The inhibitor levels used were: 1.80, 3.60, 5.40, and 7.20 g/l acetic acid (Merck); 0.61, 1.22, 1.83, and 2.44 g/l formic acid (Sigma-Aldrich); 1.72, 3.45, 5.17, and 6.89 g/l lactic acid (Sigma-Aldrich); 0.69, 1.38, 2.08, and 2.77 g/l furfural (Sigma-Aldrich); and 0.94, 1.86, 2.81, and 3.75 g/l HMF (Sigma-Aldrich). Lactic acid, although not present in high amounts in lignocellulosic hydrolysates, was also included in these experiments because it can be present at high levels in large-scale fermentations as a consequence of contamination by lactic acid bacteria.

Inhibitors were also formulated into four cocktails (A, B, C and D), by increasing the dose of each toxic compound (Table 3).

Yeast cells grown overnight at 30°C in YNB broth at 100 rpm were transferred at an inoculum concentration of 1 × 10^6^ cells/ml in 2 ml eppendorf tubes containing 0.9 ml of medium and aerobically incubated. After 40 hours of growth at 30°C, the optical density at 600 nm (OD_600 nm_) was measured. For each strain, the tolerance was evaluated as relative growth (OD value, %) by comparing the growth in the medium with and medium without the inhibitors.

### Fermentation of synthetic inhibitor cocktails

The most promising yeasts, selected on the basis of their high fermentation abilities and inhibitor tolerance, were studied for their fermentation performance in YNB supplemented with 100 g/l glucose and 50 g/l xylose, and each the four inhibitor cocktails A to D. The pH of the medium was adjusted to 4.5 after addition of inhibitors, using 5 mol/l NaOH.

The fermentations were performed under oxygen-limited conditions in 110 ml glass vessels (working volume of 100 ml) sealed with rubber stoppers and equipped with needles for carbon dioxide removal and sampling. Pre-cultures of yeast strains grown to stationary phase in YNB broth were used as inoculum. After centrifugation (5 min, 2,235 × *g*), yeast cells were added to a OD_600 nm_ value of 0.65, which corresponds to a dry cell weight (DCW) of approximately 0.25 g/l. Incubation was performed at 30°C with magnetic stirring and the fermentations were run for 96 hours under aseptic conditions. Samples for HPLC analysis were withdrawn at regular intervals. Samples of 10 ml were collected daily to determine DCW as described in the paragraph on ‘Analytical methods and calculations’.

### Fermentation of sugarcane bagasse hydrolysates

Sugarcane bagasse was provided by the South African Sugarcane Research Institute (SASRI) and its composition was determined using the standard laboratory analytical procedures for biomass analysis provided by the National Renewable Energy Laboratory (NREL; CO, USA) [46]. Accordingly, sugarcane bagasse was determined to comprise 57.6% glucan, 22.9% xylan, 3.2% arabinan, 19.2% lignin, 4.0% ash, and 6.8% extractives on a dry weight basis.

Hydrolysate was produced from sugarcane bagasse in a steam explosion plant equipped with a 19 liter reactor vessel, a collection tank, and a 40 bar electrical boiler. Sugarcane bagasse samples were milled to a uniform size of between 3.5 and 10 mm, and dried in a drying chamber to a final moisture content of 10% (w/w). Samples (1 kg) of this dried material were loaded into the steam pretreatment reactor, and treated at 200°C for 10 minutes. After the material had exploded, the hydrolysate was removed using a locally manufactured dead-end press, with the remaining solids having a moisture content of 40% (w/w). The hydrolysate was stored refrigerated at low pH (~pH 2) until use. The content of sugars and inhibitors was analyzed by HPLC.

To evaluate the fermentation performance of the selected yeasts on the sugarcane hydrolysate (SH), four different broths were used. One of the media consisted of hydrolysate that was not diluted (100% SH), while the other three broths were prepared by diluting the 100% SH to a concentration of 25%, 50% and 75% (v/v) using double-distilled water and are hereafter referred to as 25% SH, 50% SH and 75% SH, respectively. The concentrations of glucose and xylose in all SH broths were adjusted to 100 and 50 g/l, respectively. The hydrolysate was supplements with essential nutrients by the addition of 6.7 g/l YNB without amino acids. YNB broth supplemented with 100 g/l glucose and 50 g/l xylose was used as the reference medium and named 0% SH. The pH was adjusted to 4.5 with 5 mol/l NaOH, and the resulting media were filtered through a 0.45 μm membrane. Fermentations were performed as previously described in Methods - Fermentation of synthetic inhibitor cocktails. Pre-cultures of yeast strains grown to stationary phase in YNB broth were used as inocula. After centrifugation (5 minutes at 2,235 × *g*), yeast cells were added to give a final OD_600 nm_ of 0.65, which corresponds to a cell concentration of approximately 0.25 g/l DCW. For each sample collected during the fermentations, yeast cells were counted in triplicate using a Thoma chamber (depth, 0.02 mm).

### Analytical methods and calculations

DCWs were determined from 10 mL culture samples. Cells were collected after centrifugation (5 minutes at 2,235 × *g*), washed several times with deionized sterile water, and dried in an oven (80°C) to constant weight. Samples taken before and during fermentation kinetics were analyzed for content of arabinose, galactose, glucose, xylose, mannose, acetic acid, formic acid, lactic acid, furfural, HMF, and phenolics. Samples were filtered through a 0.22 μm membrane filter. and diluted prior to HPLC analysis. Monosaccharide analysis was performed with high-performance anion-exchange chromatography with pulsed amperometric detection (HPAEC-PAD). The system was equipped with a PA1 column and auto-sampler (Dionex Corporation, Sunnyvale, CA, USA). The mobile phase used was 1 mol/l NaOH at a flow rate of 1 ml/min at room temperature.

Organic acids, ethanol, furfural, and HMF were separated on an Aminex HPX-87H column (Bio-Rad, Hercules, CA, USA) at 65°C with 5 mmol/l H_2_SO_4_ used as the mobile phase, at a flow rate of 0.5 ml/min. The system (Shimadzu, Kyoto,Japan) was equipped with a refractive index detector (Shimadzu, Kyoto, Japan) and cation-H refill cartridge (Bio-Rad, Hercules, USA).

Phenolic acids and aldehydes (ferulic acid, vanillin, vanilic acid, syringic acid, syringaldehyde, and p-coumaric acid) were analyzed on a Phenomenex Luna C18 reversed phase column (Phenomenex Inc, Castel Maggiore, Italy) at 25°C with a flow rate of 0.7 ml/min. The mobile phases used for elution were 5 mmol/l trifluoroacetic acid in water (phase A) and 5 mmol/l trifluoroacetic acid in acetonitrile (phase B). Separation was carried out by gradient elution with an initial isocratic step at 5% mobile phase B for 5 minutes, increasing to 33% B over 55 minutes and then increasing to 100% B over 10 minutes. The mobile phase composition was then kept constant at 100% B for 10 min, followed by a decrease to 5% B over 15 minutes and ending with a final step of constant composition at 5% B for 5 minutes to allow equilibration. Phenolic acid and aldehyde peaks were detected with a Dionex Ultimate 3000 diode array detector (Thermo Fisher Scientific Inc. Waltham, MA, USA) at 280 nm.

The ethanol yield (*Y*_E/G_) from glucose was calculated as the highest amount of ethanol in grams formed per gram of consumed glucose (g/g). The volumetric productivity (*Q*_48h_) was based on grams of ethanol produced per liter of culture medium per hour, during the first 48 hours of fermentation (g/l/h). The specific productivity (*q*_48h_), based on the respective volumetric productivity divided by the correspondent DCW value, was also calculated. The glycerol yield was calculated as the amount of glycerol in grams formed per gram of consumed glucose (g/g). The oxygen-limited growth yield (Y_X/G_) (hereafter referred as the biomass yield) was calculated as the increase in cell mass after 72 hours, divided by the initial glucose concentration (g/g). The glucose consumption rate was considered as the glucose consumed per hour within the first 48 hours (g/l/h). Triplicate data were analyzed suing Microsoft Excel with one-way analysis of variance. *P* < 0.05 was considered significant.

## Abbreviations

DCW: Dry cell weight; HMF: 5-Hydroxymethyl-2-furaldehyde; HPAEC-PAD: High-performance anion-exchange chromatography with pulsed amperometric detection; HPLC: High-performance liquid chromatography; MNS: Must nutritive synthetic; NREL: National renewable energy laboratory; OD: Optical density; PCR: Polymerase chain reaction; Q48h: Volumetric productivity after 48 hours of incubation; q48h: Specific productivity after 48 hours of incubation; SASRI: South African sugarcane research institute; SSL: Spent sulfite liquor; YE/G: Ethanol yield; YM: Yeast and mould; YNB: Yeast nitrogen base; YPD: Yeast peptone dextrose; YX/G: Oxygen-limited growth yield.

## Competing interests

The authors declare that they have no competing interests.

## Authors’ contributions

LF participated in the planning of the study and the experimental design, carried out the yeast isolation, screening, and fermentation experiments, performed data analysis and interpretation, and drafted the manuscript. MB participated in the planning of the study, experimental design, and data analysis and interpretation, and commented on the manuscript. AT participated in the yeast isolation, screening, and fermentation experiments. EVR participated in the experimental design of the sugarcane bagasse hydrolysate fermentations, and extensively revised the manuscript. MGA participated in the sugarcane bagasse hydrolysate fermentations and related data analysis, and commented on the manuscript. WVZ participated in the planning of the study and data interpretation, and commented on the manuscript. SC participated in the planning of the study, experimental design, and data analysis and interpretation, and commented on the manuscript. All authors read and approved the final manuscript.

## Supplementary Material

Additional file 1: Table S1 Composition in terms of sugars and inhibitors of the SH studied in this work. Table reports the composition in terms of sugars and inhibitors (weak acids, furans, and phenolics) of the SH used in this study. Click here for file
