# Supplementary material for: Exploring grape marc as trove for new thermotolerant and inhibitor-tolerant Saccharomyces cerevisiae strains for second-generation bioethanol production
Source: Biotechnol Biofuels. 2013 Nov 29;6:168. doi: 10.1186/1754-6834-6-168 (PMC4176503; doi:10.1186/1754-6834-6-168)
Supplement: Additional file 1: Table S1 — Composition in terms of sugars and inhibitors of the SH studied in this work. Table reports the composition in terms of sugars and inhibitors (weak acids, furans, and phenolics) of the SH used in this study. [file 1754-6834-6-168-S1.doc]

**ADDITIONAL FILE 1**

**Table S1.** Composition of steam-exploded sugarcane bagasse hydrolysate used in this study.

| **Component** | **Concentration** |
| --- | --- |
| Cellobiose (g/L) | 1.3 |
| Glucose (g/L) | 0.5 |
| Xylose (g/L) | 7.6 |
| Arabinose (g/L) | 0.6 |
| Formic acid (g/L) | 3.0 |
| Acetic acid (g/L) | 11.2 |
| HMF (g/L) | 0.5 |
| Furfural (g/L) | 1.7 |
| Gallic acid (mg/L) | 4.0 |
| Vanillin (mg/L) | 259.6 |
| Syringaldehyde (mg/L) | 27.5 |
| Ferulic acid (mg/L) | 27.4 |
| Vanillic acid (mg/L) | 30.4 |
| Syringic acid (mg/L) | 46.5 |
| p-Coumaric acid (mg/L) | 76.3 |
